# Coffee Intake and Liver Steatosis: A Population Study in a Mediterranean Area

**DOI:** 10.3390/nu10010089

**Published:** 2018-01-15

**Authors:** Nicola Veronese, Maria Notarnicola, Anna Maria Cisternino, Rosa Reddavide, Rosa Inguaggiato, Vito Guerra, Ornella Rotolo, Iris Zinzi, Gioacchino Leandro, Mario Correale, Valeria Tutino, Giovanni Misciagna, Alberto Ruben Osella, Caterina Bonfiglio, Gianluigi Giannelli, Maria Gabriella Caruso

**Affiliations:** 1 Ambulatory of Clinical Nutrition, National Institute of Gastroenterology-Research Hospital, IRCCS “S. de Bellis”, Castellana Grotte, 70013 Bari, Italy; ilmannato@gmail.com (N.V.); annamaria.cisternino@irccsdebellis.it (A.M.C.); rosa.reddavide@irccsdebellis.it (R.R.); rosa.inguaggiato@irccsdebellis.it (R.I.); rotolornella@gmail.com (O.R.); iris.zinzi@libero.it (I.Z.); gioacchino.leandro@irccsdebellis.it (G.L.); 2 Laboratory of Nutritional Biochemistry, National Institute of Gastroenterology-Research Hospital, IRCCS “S. de Bellis”, Castellana Grotte, 70013 Bari, Italy; maria.notarnicola@irccsdebellis.it (M.N.); valeria.tutino@irccsdebellis.it (V.T.); 3 National Research Council, Neuroscience Institute, Aging Branch, 35128 Padova, Italy; 4 Clinical Trial Unit, National Institute of Gastroenterology-Research Hospital, IRCCS “S. de Bellis”, Castellana Grotte, 70013 Bari, Italy; vito.guerra@irccsdebellis.it; 5 Unit of Gastroenterology, National Institute of Gastroenterology-Research Hospital, IRCCS “S. de Bellis”, Castellana Grotte, 70013 Bari, Italy; 6 Laboratory of Clinical Pathology, National Institute of Gastroenterology-Research Hospital, IRCCS “S. de Bellis”, Castellana Grotte, 70013 Bari, Italy; mario.correale@irccsdebellis.it; 7 Scientific and Ethical Committee, University Hospital Policlinico, 70124 Bari, Italy; gmisciag@libero.it; 8 National Institute of Gastroenterology-Research Hospital, IRCCS “S. de Bellis”, Castellana Grotte, 70013 Bari, Italy; arosella@irccsdebellis.it (A.R.O.); catia.bonfiglio@irccsdebellis.it (C.B.); gianluigi.giannelli@irccsdebellis.it (G.G.)

**Keywords:** fatty liver, coffee, caffeine, ultrasound, epidemiology

## Abstract

Coffee drinking seems to have several beneficial effects on health outcomes. However, the effect on hepatic steatosis, depending on a high alcohol consumption (AFLD, alcoholic fatty liver disease) or on metabolic factors (non-alcoholic fatty liver disease, NAFLD), is still equivocal. Thus, we aimed to explore the potential association between coffee consumption and the presence and severity of hepatic steatosis in people with NAFLD or AFLD. In this cross-sectional study, coffee drinking was recorded using a semi-quantitative food frequency questionnaire, and categorized as yes vs. no and as 0, 1, 2, ≥3. The degree of fatty liver was assessed through a standardized ultrasound examination (score 0 to 6, with higher values reflecting higher severity). Liver steatosis was classified as NAFLD or AFLD on daily alcohol intake >30 g/day for men and >20 g/day for women. This study included 2819 middle-aged participants; the great majority were coffee drinkers (86.1%). After adjusting for 12 potential confounders, drinking coffee was not associated with decreased odds for NAFLD (*n* = 916) (odds ratio, OR = 0.93; 95% confidence intervals, CI: 0.72–1.20) or AFLD (*n* = 276) (OR = 1.20; 95% CI: 0.66–2.0). The consumption of coffee (categorized as yes vs. no), or an increased consumption of coffee were not associated with the presence of mild, moderate or severe liver steatosis in either NAFLD or AFLD. In conclusion, coffee intake was not associated with any lower odds of hepatic steatosis in either non-alcoholic or alcoholic forms in this large cohort of South Italian individuals.

## 1. Introduction

Coffee drinking is a very common habit in Western countries. Current research suggests that almost 85% of the American population drink at least one coffee a day for various reasons, including to improve mental alertness, concentration, and reduce fatigue [1].

It has been reported that coffee has several beneficial effects on human health. Recently, coffee consumption was reported to significantly decrease overall mortality in a large European cohort involving about 500,000 subjects followed up for 16 years [2]. Other studies have reported the beneficial effects of coffee for several medical conditions, including diabetes mellitus [3] and Parkinson’s disease [4]. Finally, coffee consumption seems to have beneficial effects for some important hepatic medical conditions, such as hepatitis C virus infection [5], fibrosis and cirrhosis [6], and hepatocellular carcinoma [7].

However, steatosis is the most common condition affecting the liver. When associated with a low alcohol intake, it is traditionally attributed to the presence of metabolic disorders, such as hypertension, dyslipidemia, obesity, and impaired glucose tolerance, which contribute to its rapidly escalating prevalence, in a picture commonly called non-alcoholic fatty liver disease (NAFLD) [8].

The effect of coffee in people with NAFLD is not unequivocally clear, as reported in a recent meta-analysis [9]. Pooling data from six studies, a higher caffeine intake was not associated with any elevated prevalence of NAFLD. Even if this work advanced our knowledge regarding the possible effect of coffee in NAFLD, only few such studies have been published. This topic, however, could be of importance, since coffee is easily available, and it could be important for clinicians to know if they would be justified in suggesting that patients with NAFLD drink more coffee in order to decrease liver steatosis as an additional therapeutic approach. Moreover, there are no data regarding the impact of coffee in alcoholic fatty liver disease (AFLD) that is the other major cause of liver steatosis worldwide.

Against this background, we aimed to explore the potential association between coffee consumption and the presence and severity of hepatic steatosis in people with NAFLD or AFLD in a large cohort of participants living in South Italy.

## 2. Materials and Methods

### 2.1. Participants

This study included women and men randomly sampled from the electoral rolls of the population of Castellana Grotte, a town in Southern Italy (Apulia region), between 2005 and 2006. Among 1942 initially contacted, 1708 (87.9%) participated in the baseline survey (MICOL III). Additionally, in the same years, 1900 young participants (between 30 and 49 years) were contacted (panel study). Among them, 1265 (66.6%) decided to participate in the study.

The Research proposal was approved by the Institutional Review Board (Ethics Committee) of IRCCS De Bellis and written informed consent was obtained from each participant before entry into the study.

### 2.2. Measurements

The survey visit consisted of the administration of a standardized questionnaire, including a validated semi-quantitative food frequency questionnaire, anthropometric measurements, a blood sample for biochemical tests, and an ultrasonographic examination. Blood sampling was performed in the morning after overnight fasting and biochemical measures were recorded using standardized methods.

For the purposes of this research, we included: (a) a smoking habit categorized as previous/current vs. never; (b) body mass index (BMI) and waist circumference, recorded by a trained physician or nurse using standardized methods; (c) blood pressure (systolic and diastolic), recorded once at the right arm; (d) daily energy and alcohol intake; (e) self-reported history of diabetes, gastric ulcer, previous cancer (more than five years beforethe interview), and acute myocardial infarction.

### 2.3. Exposure: Coffee Drinking

The main exposure in our analysis was coffee drinking recorded through the self-reported information in the semi-quantitative food frequency questionnaire. Each participant was asked if he/she had drunk coffee in the previous year, and how many cups per day. The number of coffee cups was consequently categorized as 0, 1, 2, ≥3.

### 2.4. Outcomes: Fatty Liver Disease

All the subjects underwent a standardized ultrasound examination made by two investigators using a Hitachi H21 Vision (Hitachi Medical Corporation, Tokyo, Japan). People with active chronic hepatitis (hepatitis B (HBV) or hepatitis C (HCV)), acute forms, autoimmune hepatitis, cirrhosis, or hemochromatosis were excluded from this work.

Examination of the visible liver parenchyma was performed with a 3.5 MHz transducer. A scoring system was adopted to obtain a semi-quantitative evaluation of fat in the liver [10]. The degree of liver fatty infiltration was graded according to the appearance of the liver echotexture, the hepatic echo penetration and the clarity of the hepatic blood vessels, as well as the liver diaphragm differentiation by echo amplitude.

For each criterion, a score was assigned indicating the level of fatty liver infiltration. For each criterion, a score of 2 indicated a definite positive (++) fatty liver infiltration, a score of 1 a probably positive (+) fatty liver infiltration, a score of 0 the absence of fatty liver (−). The fatty liver score ranged from 0 to 6, with higher values indicating a greater severity. Finally, liver steatosis was counted as NAFLD or AFLD, using the standard cut-offs for alcoholic liver disease [11] (>30 g/day for men, and >20 g/day for women).

### 2.5. Statistical Analysis

Mean and standard deviation were used as index of centrality and dispersion of the distributions. Student t test and chi-square distributions were used to test statistical hypotheses, with the level of statistical significance of the null hypothesis rejection set at *p* < 0.05.

The association between coffee drinking (yes vs. no) and the presence of hepatic steatosis were estimated by multivariable binary logistic regression analysis and reported as odds ratios (ORs) and 95% confidence intervals (CIs), stratified by alcohol intake. Similarly, taking exposure as the number of coffees drunk each day using people who did not drink coffee as reference, we report the ORs for those drinking 1, 2, or ≥3 coffees. A multinomial logistic regression analysis was performed to assess the association between coffee drinking (yes vs. no and as 0, 1, 2, ≥3 coffees per day) and the severity of hepatic steatosis (from 1 to 6). Again, these data are reported in people affected by NAFLD or AFLD, respectively.

Factors that were significantly different between coffee drinkers or not or significantly associated with the presence of hepatic steatosis at univariate analysis (*p* value < 0.05) were included. Multi-collinearity among covariates was assessed using the variance inflation factor (VIF), with a score of 2 causing the exclusion of a variable. For this reason, the BMI was excluded due to having a high collinearity with waist circumference (VIF = 6.98).

All the analyses were performed using the SPSS 21.0 for Windows (SPSS Inc., Chicago, IL, USA).

## 3. Results

### 3.1. General Characteristics

The two cohorts initially included 2973 participants (1265 from the panel study and 1708 from the MICOL III). After excluding 135 with no data regarding coffee intake and another 19 with no data regarding liver echography, 2819 participants were finally included. These participants had a mean age of 54.5 ± 15 years (range: 30–89) and were prevalently males (57.1%).

The study subjects’ coffee drinking characteristics (no vs. yes) are shown in Table 1. There were 2428 coffee drinkers, representing 86.1% of the whole population. These subjects consumed an average of 2.2 cups of coffee per day.

When compared to people not drinking coffee (*n* = 391), coffee drinkers were significantly younger, male, and smoked (*p* < 0.0001 for all the comparisons) (Table 1). Conversely, no differences emerged in terms of anthropometric characteristics and blood pressure values. People habitually drinking coffee reported a significantly higher daily energy intake, including a higher alcohol intake. Coffee drinkers had a significantly lower prevalence of diabetes (*p* = 0.001) and acute myocardial infarction (*p* = 0.01) than those not drinking coffee (Table 1).

Finally, no differences in terms of NAFLD (36.8% vs. 31.8% in non vs habitual coffee drinkers, *p* = 0.28, respectively) or AFLD (6.6% vs. 10.3%, *p* = 0.38) emerged.

### 3.2. Coffee Drinking and Liver Steatosis in NAFLD

Table 2 shows the association between coffee drinking and the presence and severity of fatty liver, in people reporting a low alcohol intake. After adjusting for 12 potential confounders, drinking coffee was not associated with any decreased odds of having NAFLD (*n* = 916) (OR = 0.93; 95% CI: 0.72–1.20; *p* = 0.28). Taking those not consuming coffee as a reference, people that drank 3 or more cups of coffee did not have decreased odds for liver steatosis (OR = 0.97; 95% CI: 0.71–1.32; *p* = 0.84) (Table 2, first column). Similar findings were evident for those drinking 1 or 2 coffees each day.

We further analyzed the possible association between coffee drinking and liver steatosis severity. The consumption of coffee (categorized as yes vs. no) was not associated with any presence of mild, moderate or severe liver steatosis, nor was an increased consumption of coffee associated in patients with NAFLD (Table 2). 

### 3.3. Coffee Drinking and Liver Steatosis in AFLD

Table 3 reports the association between coffee drinking and the presence and severity of fatty liver in people declaring a high alcohol intake. In the multivariable analysis, drinking coffee was not associated with any decreased odds of having AFLD (*n* = 276) (OR = 1.20; 95% CI: 0.66–2.20; *p* = 0.57). Similar to NAFLD, taking those not consuming coffee as a reference, people that drank 3 or more cups of coffee did not have decreased odds for liver steatosis (OR = 1.13; 95% CI: 0.58–2.21; *p* = 0.68) (Table 3, first column).

Drinking coffee (categorized as yes vs. no) or a greater consumption of coffee were not associated with the presence of mild, moderate or severe liver steatosis, as fully reported in Table 3.

## 4. Discussion

In our study, which involved almost 3000 South Italian participants, coffee drinking was not associated with any decreased odds for hepatic steatosis (in either non-alcoholic or alcoholic forms). Moreover, the intake of coffee was not associated with the severity of liver steatosis. Altogether our findings suggest that is unlikely that coffee drinking might be protective against the onset of hepatic steatosis.

In pre-clinical studies, caffeine seems to be able to block the progression of liver steatosis, through several pathways. Firstly, caffeine showed antioxidant properties by restoring the redox equilibrium at lipid peroxidation and glutathione peroxidase (GPx) levels [12]. This effect was also confirmed in human beings [13]. Secondly, caffeine seems to be able to promote the blockade of transforming growth factor-β (TGF-β) [12] expression and of its downstream inductor connective tissue growth factor [14]. Thirdly, caffeine might inhibit hepatic stellate cells because of a blockade of α-smooth muscle actin expression [15]. All these factors (oxidative stress, TGF-β, hepatic stellate cells) seem to be able to promote not only fibrosis, but also the progression from normal liver to hepatic steatosis [16,17,18]. Finally, coffee is rich in polyphenols, which are potent anti-oxidants and can further contribute to preventing hepatic steatosis and the progression from steatosis to more severe forms [19,20]. Thus, from a hypothetical point of view, caffeine could prevent or reverse the steatosis afflicting the liver. However, contrary to these hypotheses, we failed to find any significant association between coffee intake and liver steatosis (presence or severity). Even if we do not fully understand the reasons for these non-significant findings, we can hypothesize that the intake administered to laboratory animals in laboratory that could stop the progression of hepatic steatosis is too high for human beings, at something like 6–7 coffees each day [8].

Other studies have reported a possible association between coffee intake and the presence and severity of NAFLD. In the NHANES (National Health and Nutrition Examination Survey) study, 1782 cases with NAFLD were compared with 16,768 controls, finding that a higher coffee intake was associated with a lower prevalence of NAFLD, also after adjusting for potential confounders. However, it should be noted that in this study, NAFLD was diagnosed only on liver enzymes and that the effect was clinically small (OR = 0.999, 95% CI: 0.999–1.00) [21]. More recently, a large meta-analysis including four cross-sectional and two case-control studies with a total of 20,064 subjects failed to find any significant association between a higher coffee intake and the presence or severity of NAFLD, which was in agreement with our findings [9]. Thus, further epidemiological studies, particularly with a longitudinal design, are needed.

Our study has some strengths worthy of note. Firstly, to the best of our knowledge, it is the first to assess a possible association between coffee intake and hepatic steatosis in alcoholic fatty liver disease while, so far as we know, only studies assessing NAFLD are available. AFLD is a common complication in heavy alcohol drinkers, and therefore, knowing if (or not) caffeine is able to avoid/delay this condition is of importance. Secondly, we assessed not only the presence of hepatic steatosis, but also of the severity, again finding no association. The meta-analysis cited above reported only two studies showing that a higher caffeine intake was associated with a decreased progression of hepatic fibrosis [9]. However, the sample size was very small, limiting these findings [9]. Our study including about 2500 participants did not find any significant association, suggesting that such an association is unlikely.

The findings of our study should be interpreted taking into account its limitations. Firstly, the cross-sectional design of the study makes it difficult to understand the direction of the relationship between fatty liver disease severity and coffee intake. Secondly, we did not perform a liver biopsy, which is considered the gold standard for the diagnosis of fatty liver. The sensitivity of ultrasound in detecting hepatic steatosis does, in fact, range widely (60–94%) depending on the population chosen for the study [22]. Thirdly, the number of people with a severe grade of either AFLD/NAFLD was small, and this could affect our results. Finally, we used coffee cups as estimates of caffeine intake, but this estimate can be significantly different when compared to cup sizes in other nations, such as the USA, in terms of quantity of caffeine introduced.

## 5. Conclusions

In conclusion, our study suggests that coffee intake was not associated with any lower odds for hepatic steatosis in either non-alcoholic or alcoholic forms in this large cohort of South Italian individuals. Since other data suggested that the consumption of coffee could help in the prevention of hepatic steatosis, future longitudinal and interventional studies using caffeine as a nutraceutical component, ideally without the important side effects on the cardiovascular system, are warranted.

## Figures and Tables

**Table 1:** Demographic and other characteristics of the study subjects by coffee drinking.

Variables	Coffee		
	No	Yes	*p*^1^
	(*n* = 391)	(*n* = 2428)	
Number of cups	-	2.2 ± 1.2	-
Age (years)	59.7 ± 17.6	53.7 ± 14.4	<0.0001
Females (%)	52.4	41.4	<0.0001
Smokers (previous/current) (%)	26.3	45.6	<0.0001
BMI (kg/m²)	28.6 ± 5.1	28.7 ± 5.3	0.82
Waist circumference (cm)	92.1 ± 13.0	93.0 ± 13.4	0.24
Systolic blood pressure (mmHg)	124.6 ± 20.1	122.9 ± 19.7	0.13
Diastolic blood pressure (mmHg)	75.2 ± 9.8	74.6 ± 10.3	0.29
Daily energy intake (Kcal)	2084 ± 780	2276 ± 833	<0.0001
Daily alcohol intake (g)	10.4 ± 18.5	17.3 ± 22.7	<0.0001
Diabetes (%)	14.1	8.4	0.001
Gastric ulcer (%)	8.7	9.3	0.78
Previous cancer (%)	3.6	4.0	0.89
Acute myocardial infarction (%)	5.1	2.6	0.01
Fatty liver (%)	43.4	42.1	0.62
Non-alcoholic fatty liver * (%)	36.8	31.8	0.28
Alcoholic fatty liver ° (%)	6.6	10.3	0.38

Note: Values are reported as mean ± SD (for continuous variables), or % for categorical ones; ^1^ *p* values were calculated using the independent *t*-test for continuous variables and Chi-square test for categorical ones; * Alcohol consumption ≤30 g/day (men), ≤20 g/day (women); ° Alcohol consumption >30 g/day (men), >20 g/day (women). BMI: body mass index.

**Table 2:** Association between coffee consumption and liver steatosis in people with Non-alcoholic Fatty Liver Disease (NAFLD) *.

Coffee Parameters	Categories	Liver Steatosis Score ^2^							
		NAFLD ^1^	1	2	3	4	5	6	≥3
		(*n* = 916)	(*n* = 239)	(*n* = 154)	(*n* = 156)	(*n* = 199)	(*n* = 34)	(*n* = 134)	(*n* = 523)
Coffee consumption	Yes	0.93 (0.72–1.20)	1.05 (0.70–1.58)	1.21 (0.76–1.94)	1.08 (0.67–1.77)	1.11 (0.71–1.73)	0.97 (0.35–2.65)	0.92 (0.52–1.63)	1.35 (0.67–2.73)
Number of coffees	1	0.89 (0.67–1.19)	0.92 (0.56–1.51)	1.13 (0.64–2.00)	0.96 (0.54–1.71)	1.07 (0.63–1.80)	1.74 (0.45–6.70)	1.03 (0.54–1.99)	1.02 (0.73–1.43)
	2	0.94 (0.70–1.25)	0.85 (0.56–1.29)	0.73 (0.43–1.24)	0.95 (0.59–1.54)	0.97 (0.62–1.52)	1.18 (0.33–4.20)	1.16 (0.67–1.99)	0.97 (0.69–1.369
	≥3	0.97 (0.71–1.32)	0.82 (0.56–1.22)	1.11 (0.70–1.76)	0.74 (0.46–1.18)	0.92 (0.61–1.40)	3.16 (1.10–9.05)	1.20 (0.73–2.00)	0.96 (0.67–1.36)

Notes: * Alcohol consumption: Males (≤30 g/day); Females (≤20 g/day); ^1^ Logistic regression analysis was applied; ^2^ multinomial logistic regression analysis was applied. In both analyses, people without evidence of liver steatosis were taken as reference. The data are reported as odds ratios (ORs) and 95% confidence intervals (CIs) after adjusting for age, sex, smoking status (current/previous vs. never), presence of diabetes, gastric ulcer, cancer, acute myocardial infarction (all yes vs. no); waist circumference, systolic and diastolic blood pressure, daily energy and alcohol intake (all as continuous variables).

**Table 3:** Association between coffee consumption and liver steatosis in people with Alcoholic Fatty Liver Disease (AFLD) *.

Coffee Parameters	Categories	Liver Steatosis Score ^2^							
		AFLD ^1^	1	2	3	4	5	6	≥3
		(*n* = 276)	(*n* = 75)	(*n* = 35)	(*n* = 62)	(*n* = 61)	(*n* = 11)	(*n* = 32)	(*n* = 166)
Coffee consumption	Yes	1.20 (0.66–2.20)	1.19 (0.50–2.84)	0.59 (0.13–2.72)	0.78 (0.28–2.18)	0.52 (0.15–1.85)	Too few subjects	1.19 (0.36–3.93)	1.35 (0.67–2.73)
Number of coffees	1	1.04 (0.54–1.99)	1.09 (0.40–2.93)	0.75 (0.14–3.96)	1.08 (0.33–3.51)	0.52 (0.14–2.03)	Too few subjects	1.23 (0.31–4.83)	1.24 (0.58–2.64)
	2	1.42 (0.75–2.68)	0.73 (0.34–1.54)	1.20 (0.41–3.52)	1.26 (0.54–2.93)	0.92 (0.41–2.05)	Too few subjects	0.82 (0.27–2.56)	1.54 (0.74–3.23)
	≥3	1.13 (0.58–2.21)	1.06 (0.54–2.07)	1.54 (0.59–3.99)	1.73 (0.80–3.73)	1.08 (0.52–2.27)	Too few subjects	1.24 (0.47–3.30)	1.23 (0.56–2.69)

Note: * Alcohol consumption: Males (>30 g/day); Females (>20 g/day); ^1^ Logistic regression analysis was applied; ^2^ multinomial logistic regression analysis was applied. In both analyses, people without evidence of liver steatosis were taken as reference. The data are reported as odds ratios (ORs) and 95% confidence intervals (CIs) after adjusting for age, sex, smoking status (current/previous vs. never), presence of diabetes, gastric ulcer, cancer, acute myocardial infarction (all yes vs. no); waist circumference, systolic and diastolic blood pressure, daily energy and alcohol intake (all as continuous variables).
